# Survival rate and peri-implant evaluation of immediately loaded dental implants in individuals with type 2 diabetes mellitus: a systematic review and meta-analysis

**DOI:** 10.1007/s00784-021-04154-6

**Published:** 2021-09-29

**Authors:** Carlos Alexandre Soares Andrade, João Lucas Carvalho Paz, Gabriel Simino de Melo, Nour Mahrouseh, Alessandro Lourenço Januário, Lucas Raineri Capeletti

**Affiliations:** 1 Faculty of Medicine, Department of Public Health and Epidemiology, University of Debrecen, Debrecen, Hajdú-Bihar, Hungary; 2 Department of Periodontology and Implant Dentistry, Universidade Federal de Uberlândia (UFU), Uberlândia, Minas Gerais Brazil; 3 Faculty of Medicine and Dentistry, Postgraduate Department, São Leopoldo Mandic, Campinas, São Paulo, Brazil; 4 Department of Periodontology and Implant Dentistry, Instituto Aria, Brasília, Distrito Federal Brazil; 5 Department of Dentistry, Universidade Federal de Goiás (UFG), Goiânia, Goiás Brazil

**Keywords:** Immediate Dental Implant Loading, Diabetes Mellitus, Edentulism

## Abstract

**Objectives:**

To evaluate the survival rate, success rate, and peri-implant biological changes of immediately loaded dental implants (ILs) placed in type 2 diabetic patients (DM2).

**Materials and methods:**

The present study was registered on PROSPERO and followed the PRISMA checklist. The search was performed by the first reviewer in January 2021. The electronic databases used were MEDLINE via PubMed, Cochrane, BVS, Web of Science, Scopus, LIVIVO, and gray literature. The risk of bias analysis was performed using an instrument from the Joanna Briggs Institute.

**Results:**

A total of 3566 titles and abstracts were obtained. The qualitative synthesis included 7 studies, while the quantitative synthesis included 5 studies. The meta-analysis of IL in individuals with DM2 compared to nondiabetic individuals showed no significant difference among the groups regarding the survival rate of dental implants (RR = 1.00, 95% CI 0.96–1.04; *p* = 0.91; *I*^2^ = 0%), even if the patient had poor glycemic control (RR = 1.08, 95% CI 0.87–1.33; *p* = 0.48; *I*^2^ = 70%). Meta-analysis of marginal bone loss in IL compared to conventional loading in DM2 patients also showed no significant difference (mean difference =  − 0.08, 95% CI − 0.25–0.08; *p* = 0.33; *I*^2^ = 83%).

**Conclusions:**

Type 2 diabetes mellitus does not seem to be a risk factor for immediately loaded implants if the glycemic level is controlled, the oral hygiene is satisfactory, and the technical steps are strictly followed.

Clinical relevance

Rehabilitation in diabetic individuals is more common due to the highest prevalence of edentulism in this population. It is essential to establish appropriate protocols for loading dental implants.

**Supplementary Information:**

The online version contains supplementary material available at 10.1007/s00784-021-04154-6.

## Introduction

Diabetes mellitus is a highly prevalent metabolic syndrome and is classified by World Health Organization (WHO) as the 6th leading cause of death in the world [1, 2]. The International Diabetes Federation (IDF) estimates that 374 million people live with the disease worldwide, and the projections for 2045 indicate that the prevalence of diabetes could increase to 548 million people [3]. The COVID-19 pandemic situation has demonstrated that compared to nondiabetic patients, diabetic patients have higher mortality and more severe outcomes when infected with SARS-CoV-2 [4]. Type II diabetes mellitus (DM2) is the most prevalent type of diabetes, accounting for 85 to 95% of diabetic individuals [3]. DM2 can lead to long-term damage and systemic complications, such as neuropathy, structural damage to blood vessels, poor healing processes, affected peripheral microcirculation, arterial hypertension, and unsatisfactory immune responses [5].

The prevalence of edentulism in DM2 patients is extremely high due to various oral manifestations, such as periodontitis, root caries, and endodontic disease [6–8]. Thus, with the increased incidence of DM2 worldwide, there is also a higher demand for satisfactory solutions in oral rehabilitation to improve the quality of life of this population [9]. Recent systematic reviews regarding dental implants in diabetic patients have shown that DM2 is associated with a higher risk of developing peri-implant diseases or complications [10–13]. The success rate in the dental implant field widely differs, depending on the reference. Peri-implant parameters such as bleeding on probing (BOP), pocket depth (PD), and marginal bone loss (MBL) have been used to measure the success rate of dental implants. The survival rate is usually assessed by osseointegration—if the dental implant is still in the mouth cavity or if it was removed [14].

There is insufficient evidence in the literature on the survival and success of advanced dental implant techniques in DM2 patients. Immediate loading of dental implants (ILs) is thought to decrease the patient’s rehabilitation treatment time. The placement of the prosthetic element occurs within 72 h after dental implant surgery [15]. This is considered a safe technique in cases with a good level of primary stability, bone availability, and favorable peripheral tissue conditions. One of the requirements for using this technique is the absence of systemic diseases affecting osseointegration therapy, as it is more challenging to heal IL compared to cases of conventional loading [13]. DM2 is a well-known metabolic disorder that may affect the osseointegration of IL due to the deficient process of bone remodeling caused by the formation of advanced glycation end products (AGEs) [16, 17]. Optimal osteoblast activity and minimal bone resorption occur under controlled glycemia and low levels of AGEs, mostly when the osseointegration process occurs under functional occlusal loading [17, 18]. Furthermore, chronic hyperglycemia can worsen the peri-implant soft and hard tissue healing process of IL due to compromised vascularization, oral tissue necrosis, delayed healing, and predisposition to local infections [19–21]. Although several studies have used IL in compromised healthy patients, a systematic review identifying and summarizing the findings of these studies in DM2 individuals has not yet been published. The aim of the present systematic review and meta-analysis is to provide evidence regarding the survival rate and success parameters (BOP, PD, and MBL) of IL in DM2 by answering the following questions:

1. “Is there any difference in the survival or success rate of immediately loaded dental implants in individuals with DM2 compared to that of nondiabetic patients?”
2. “Is there any difference in the survival or success rate of immediately loaded dental implants in individuals with uncontrolled DM2 compared to that of nondiabetic patients?”
3. “Do immediately loaded dental implants have worse peri-implant outcomes than conventional loaded dental implants in individuals with DM2?”

## Methodology

### Protocol and registration

The present systematic review and meta-analysis were conducted following the Preferred Reporting Items for Systematic Reviews and Meta-Analyses (PRISMA) checklist [22]. The group of authors created the protocol, and the first reviewer registered it on the International Prospective Register of Systematic Reviews (PROSPERO). The study was started after protocol approval by the identification number CRD42021223736.

### Research question and PICO

The PICO model was followed to formulate the following three research questions:

- Participants/population: The population in the present study included individuals diagnosed with type 2 diabetes mellitus (DM2) who were treated with dental implant placement, and the restoration was immediately loaded (IL). Data about uncontrolled DM2 were recorded separately in cases in which the study reported it. The excluded population comprised studies in which the type of diabetes was not reported or in which data regarding different types of diabetes were merged.
- Intervention: The indispensable intervention was the placement of immediately loaded dental implants (ILs). The size and number of dental implants placed were not delimited. The following types of restorations were considered: single teeth, partial denture, overdenture, full mouth denture, and/or the All-on-Four prosthesis.
- Comparator(s)/control: Two different comparators were considered controls, including nondiabetic individuals who received IL and/or conventional loaded dental implants (CLs) in DM2.
- Outcomes: Survival, success rate, and/or peri-implant health status were considered the outcomes of the study. The following biologic peri-implant measures were considered to assess the success of dental implants: BOP, PD, and MBL.

### Eligibility criteria

The following types of studies were eligible for this systematic review: (a) clinical trials, cohort studies, case–control, cross-sectional, or case series. (b) Studies reporting the survival or success rate of immediately loaded dental implants in human individuals with DM2. (c) Studies reporting peri-implant measurements, such as BOP, PD, and MBL, of IL in DM2 patients. No limit on the publication date was considered. The exclusion criteria were (a) systematic reviews, literature reviews, letters, editorials, books, in vitro studies, animal studies, and case reports; (b) studies in which the alphabet was non-Latin; (c) studies without a DM2 group or that did not report which type of diabetes was analyzed; and (d) studies that merged data regarding IL or DM2 were also excluded.

### Search strategy and study selection

The search was performed by the first reviewer on 04 January 2021. The electronic databases used were MEDLINE via PubMed, Cochrane, BVS, Web of Science, Scopus, and LIVIVO. In addition, the gray literature, dissertations, and theses were consulted: ProQuest, OpenGray, Google Scholar, and manual search of references list from included articles. The following search terms were used to create the search strategy: (“diabetes Mellitus” OR “hyperglycemia” OR “diabetic patients” OR diabetic OR “systemic diseases”) AND (“dental implants” OR “dental implant” OR “immediate implant” OR “implant placement” OR “immediate implants” OR “immediately loaded” OR “advanced implant” OR “implant placement”). The search terms were adapted for each of the databases and gray literature. Appendix 1 shows the specific search strategy for each database.

All titles and abstracts were downloaded from the databases and uploaded to *RAYYAN QCRI®* (Qatar Computing Research Institute, Qatar). After the removal of duplicates on the same software, two blinded independent reviewers screened the titles and abstracts based on the eligibility criteria. The reasons for inclusion or exclusion were recorded so that it was possible to further discuss them. In cases of disagreement between the two reviewers, a decision was reached by meeting with and consulting the expert. The included studies were retrieved, and the two reviewers proceeded with full-text analysis. Studies that did not fit the eligibility criteria were excluded, and the exclusion justification was indicated.

### Data extraction

In the next phase, the full-text articles that were included had their data extracted in an Excel table based on the JBI Manual for Evidence Synthesis [23]. The first reviewer performed the data extraction, and the second reviewer independently cross-checked the table to consider if the important data were reported. The extraction table was created considering the following information of each included study: (a) study details—first author, publication year, and journal; (b) study method—aims of the study, country, setting, study design, and follow-up; (c) subject characteristics—sample size, groups, age range, gender, glycemic control, and the number of implants placed; and (d) results—survival rate, success rate, MBL, BOP, and PD. In addition, technical information about the placement implant surgery and the loading protocol was extracted.

### Risk of bias

The risk of bias analysis was performed using the instrument of the Joanna Briggs Institute (Critical Appraisal Tools). The first reviewer independently answered the questionnaires of each type of study. The second reviewer independently cross-checked the answers, and any disagreements were resolved by meeting with the expert and the systematic review coordinator. Review Manager 5.3 (The Cochrane Collaboration, Copenhagen, Denmark) was the chosen software to create the figures. Studies with answers that were less than 49% “yes” were considered to have a high risk of bias. Studies with “yes” responses between 50 and 69% were considered to have a moderate risk of bias. Studies with more than 70% of “yes” answers in the questionnaire were considered to have a low risk of bias.

### Statistical analysis

Cohen’s kappa coefficient (*κ*) was used to calculate the agreement rate between the first and second reviewers in the first phase of study selection (title and abstract screening). The meta-analysis was performed if more than one study, containing at least one control group, provided homogeneous information regarding a specific topic. Three meta-analyses were conducted in the Review Manager 5.3 software (The Cochrane Collaboration, Copenhagen, Denmark): (a) IL survival in DM2 compared to nondiabetic individuals; (b) IL survival in uncontrolled DM2 compared to nondiabetic individuals; and (c) MBL of IL compared to CL in individuals with DM2 after 12 months. The number of surviving implants was pooled with weight mean differences (WMDs) to perform the meta-analysis (a) and (b). The third meta-analysis was performed based on MBL, which is a continuous variable. For this reason, the mean differences (MD) of MBL values in millimeters were considered. The three meta-analysis outcomes were measured in a 95% confidence interval (CI). The chi-square test (*p* < 0.05) and I-square index (*I*^2^) were chosen as measurements to evaluate the statistical heterogeneity and the magnitude of the inconsistency, respectively. The inconsistency was considered high if the *I*^2^ value was above 50% and low if the value was below 25%.

## Results

### Literature search and study selection

**Fig. 1:**
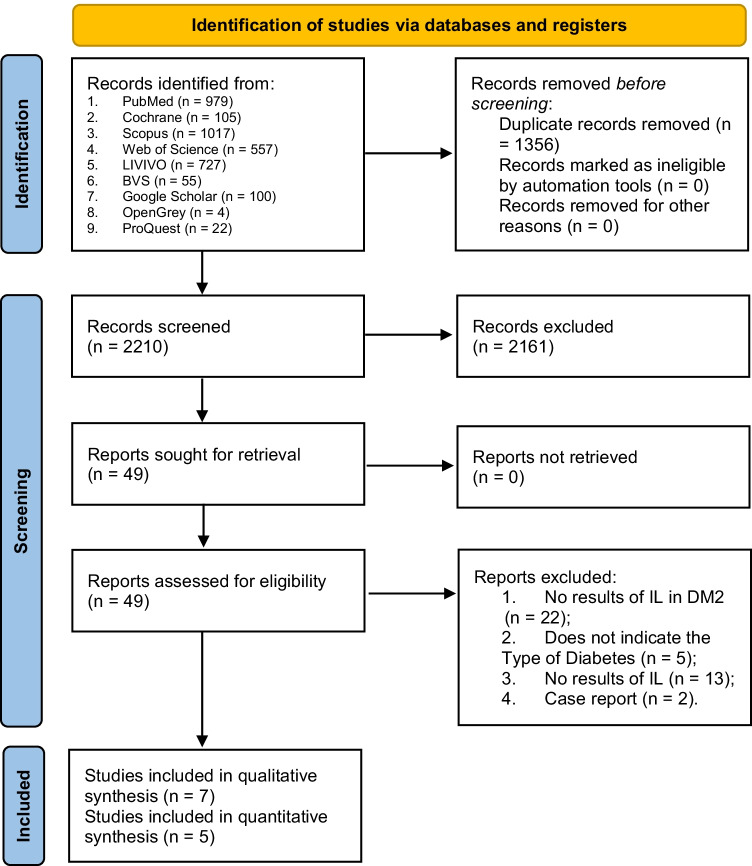
Preferred Reporting Items for Systematic Reviews and Meta-Analyses (PRISMA) flowchart 2020 describing the study selection process [22]

The searches through the databases and gray literature provided a total of 3566 titles and abstracts of studies that were downloaded and uploaded to the RAYYAN QCRI platform®. After the removal of duplicates, a total of 2210 titles and abstracts were available for screening evaluation. Two reviewers used the eligibility criteria to select a total of 49 studies for full-text analysis. The Cohen’s kappa coefficient for this phase of selection was 0.87, which is considered an “almost perfect agreement” between the two reviewers. The third reviewer was consulted to resolve disagreements between the two abstracts. Among the 49 articles, only 7 studies were eligible for qualitative analysis, and 5 of them met the criteria for quantitative analysis (meta-analysis) [16, 17, 19, 24–27]. A total of 42 studies were excluded from the full-text analysis, and the reasons were mostly due to the absence of results regarding IL in DM2. The PRISMA flow diagram shows all the phases of study selection, including the number of articles included and excluded in each section (Fig. 1) [22].

### Study characteristics

The countries in which the 7 included studies were conducted were Portugal, Lebanon, Spain, Saudi Arabia, Egypt, and Romania [16, 17, 19, 20, 24, 26, 27]. All the studies were published in the English language between 2008 [24] and 2020 [26]. Five studies were longitudinally observational [16, 19, 24, 26, 27], and two studies were clinical trials [17, 20]. Three studies were conducted in private clinics [16, 24, 27], two were conducted in healthcare centers [17, 20], and two studies did not report the setting [19, 26]. Most included studies had a follow-up ranging from 6 to 24 months [16, 17, 19, 20, 26]. One study followed the patients for 5 years [27], and another study had a 12-year follow-up [24]. The sample size differed between the seven studies, with a minimum of 4 patients [26] and a maximum of 108 patients [19]. The number of placed implants in each study varied from 16 [26] to 352 [27]. One study did not report the type of restoration used in oral rehabilitation [24]. The other six studies comprised the following types of prostheses: single teeth [16, 17, 27], partial dentures [27], overdentures [20], and All-on-Four prostheses [26] (Table 1). All 7 studies reported technical information regarding implant placement surgery. A few studies also provided detailed data about prosthetic loading and oral hygiene (Table 2).

**Table 1:** Main characteristics of the *n* = 7 included studies

	Study	Country	Journal	Setting	Type of study	Follow-up	Groups	Gender and age	Glycemic control	Total implants (*n*)	Failure incidence (*n*)	Type of implant-supported prosthesis	Survival rate (%)	Success rate (%)	Marginal bone loss (MBL or CBL)	Bleeding on probing (BOP)	Probing depth (PD)
1	Tawil et al. [24]	Lebanon	*The International Journal of Oral & Maxillofacial Implants*	Private periodontal practice	Prospective study	1 to 12 years	Control group: 45 patients (non DM2)Group 1: 22 patients (well-controlled DM2)Group 2: 22 patients (fairly well-controlled DM2)Group 3: 1 patient (uncontrolled DM2)	DM2: 12 female and 33 male (64.7 [43–84])Control group: 21 female and 24 male (59.6 [29–85])	Group 1: < 7% HbA1cGroup 2: 7–9% HbA1cGroup 3: > 9% HbA1c	Control group: 59DM2 group: 58	Control group: 1 implant/patientDM2 group: zero	N/A	100%		N/A		
2	Aguilar-Salvatierra et al. [16]	Spain	*Clinical Oral Implants Research*	Private practice	Prospective study	12 to 24 months	Group 1: 22 patients (non DM2)Group 2: 30 patients (controlled DM2)Group 3: 22 patients (uncontrolled DM2)	Group 1: 15 female and 18 male (59 ± 2.3)Group 2: 17 female and 13 male (57 ± 3.8)Group 3: 9 female and 13 male (61 ± 1.9)	Group 1: < 6% HbA1cGroup 2: 6.1–8% HbA1cGroup 3: 8.1–10% HbA1c	Group 1: 33Group 2: 30Group 3: 22	Group 1: zeroGroup 2: 1 implant/patientGroup 3: 3 implants/patients	Single immediate dental implant placement with immediate loading	Group 1 12 m: 100%Group 1 24 m: 100%Group 2 12 m: 100%Group 2 24 m: 96.6%Group 3 12 m: 95.4%Group 3 24 m: 86.3%	N/A	MBL group 1 6 m: 0.51 ± 0.19MBL group 1 12 m: 0.64 ± 0.23MBL group 1 24 m: 0.72 ± 0.27MBL group 2 6 m: 0.71 ± 0.31MBL group 2 12 m: 0.86 ± 0.25MBL group 2 24 m: 0.98 ± 0.27MBL group 3 6 m: 1.33 ± 0.29MBL group 3 12 m: 1.54 ± 0.43MBL group 3 24 m: 1.92 ± 0.38	Group 1 6 m: 0.36 ± 0.06Group 1 12 m: 0.39 ± 0.04Group 1 24 m: 0.44 ± 0.07Group 2 6 m: 0.41 ± 0.04Group 2 12 m: 0.45 ± 0.07Group 2 24 m: 0.51 ± 0.05Group 3 6 m: 0.59 ± 0.07Group 3 12 m: 0.65 ± 0.06Group 3 24 m: 0.74 ± 0.05	PD ≥ 4 mm group 1 6 m: 2.43 ± 0.25PD ≥ 4 mm group 1 12 m: 2.60 ± 0.18PD ≥ 4 mm group 1 24 m: 2.67 ± 0.14PD ≥ 4 mm group 2 6 m: 2.54 ± 0.32PD ≥ 4 mm group 2 12 m: 2.66 ± 0.27PD ≥ 4 mm group 2 24 m: 2.79 ± 0.24PD ≥ 4 mm group 3 6 m: 3.43 ± 0.23PD ≥ 4 mm group 3 12 m: 3.57 ± 0.37PD ≥ 4 mm group 3 24 m: 3.68 ± 0.48
3	de Araújo et al. [27]	Portugal	*Journal of Oral Rehabilitation*	Private clinic	Retrospective cohort study	1 and 5 years	Group 1: 6 patients (well-controlled DM1)Group 2: 64 patients (well-controlled DM2)	33 female37 male59 (41–80)	Fasting plasma glucose > / = 7.0 mmol/l (126 mg/dl) or 2 h plasma glucose > / = 11.1 mmol/l (200 mg/dl)	DM1 and DM2: 352	DM2 group: 9 implants/5 patients	Single teeth, partial and full-arch	90.5%	N/A	1 year: 0.79 mm (0.59–1.00 mm)5 years: 1.45 mm (1.09–1.82 mm)	N/A	
4	Al-Amri et al. [17]	Saudi Arabia	*Clinical Oral Implants Research*	Oral healthcare center in Riyadh	Randomized clinical study	6, 12, and 24 months	Group 1: systemically healthyGroup 2: controlled DM2Group 3: uncontrolled DM2	90 maleGroup 1:48.5 (45–52)Group 2: 50.1 (46–55)Group 3: 50.5 (45–59)	Group 1: < 6% HbA1cGroup 2: 6.1–8% HbA1cGroup 3: 8.1–10% HbA1c	Group 1: 30Group 2: 30Group 3: 31	Zero	Single crown	100%	N/A	MBL group 1 6 m: 0.33 ± 0.1MBL group 1 12 m:: 0.45 ± 0.06MBL group 1 24 m: 0.46 ± 0.16MBL group 2 6 m: 0.52 ± 0.02MBL group 2 12 m: 0.54 ± 0.12MBL group 2 24 m: 0.58 ± 0.15MBL group 3 6 m: 0.55 ± 0.06MBL group 3 12 m: 0.57 ± 0.07MBL group 3 24 m: 0.59 ± 0.2	Group 1 6 m: 0.42 ± 0.05Group 1 12 m: 0.4 ± 0.02Group 1 24 m: 0.4 ± 0.06Group 2 6 m: 0.63 ± 0.06Group 2 12 m: 0.6 ± 0.04Group 2 24 m: 0.62 ± 0.07Group 3 6 m: 0.71 ± 0.05Group 3 12 m: 0.63 ± 0.02Group 3 24 m: 0.62 ± 0.05	PD ≥ 4 mm group 1 6 m: 2 ± 0.5PD ≥ 4 mm group 1 12 m: 1.9 ± 0.04PD ≥ 4 mm group 1 24 m: 1.6 ± 0.05PD ≥ 4 mm group 2 6 m: 2.5 ± 0.18PD ≥ 4 mm group 2 12 m: 2.3 ± 0.26 PD ≥ 4 mm group 2 24 m: 2.3 ± 0.15PD ≥ 4 mm group 3 6 m: 3.3 ± 0.21PD ≥ 4 mm Group 3 12 m: 2.4 ± 0.35PD ≥ 4 mm group 3 24 m: 2.3 ± 0.62
5	Al-Amri et al. [19]	Saudi Arabia	*Journal of Oral Rehabilitation*	N/A	Retrospective study	12 and 24 months	Group 1: 55 patients ILGroup 2: 53 patients CL	108 maleGroup 1: 50.6 + 2.2Group 2: 51.8 + 1.7	Group 1 (12 months): 5.4% HbA1c (4.8–5.5%)Group 1 (24 months): 5.1% HbA1c (4.7–5.2%)Group 2 (12 months): 5.1% HbA1c (4.7–5.4%)Group 2 (24 months): 4.9% HbA1c (4.5–5.2%)	Group 1: 55Group 2: 53	Zero	Single crown	100%		CBL M group 1 12 m: 0.5CBL D group 1 12 m: 0.6CBL group 1 12 m: 0.55CBL M group 2 12 m: 0.6CBL D Group 2 12 m: 0.52CBL group 2 12 m: 0.56CBL M group 1 24 m: 0.57CBL D group 1 24 m: 0.61CBL group 1 24 m: 0.58CBL M group 2 24 m: 0.67CBL D group 2 24 m: 0.62CBL group 2 24 m: 0.64	Group 1 Max 12 m: 12.5 ± 2.5 (%)Group 1 Mand 12 m: 16.6 ± 3.7 (%)Group 2 Max 12 m: 13.4 ± 0.3 (%)Group 2 Mand 12 m: 16.5 ± 2.2 (%)Group 1 Max 24 m: 10.5 ± 0.5 (%)Group 1 Mand 24 m: 10.1 ± 0.2 (%)Group 2 Max 24 m: 10.2 ± 0.3 (%)Group 2 Mand 24 m: 9.1 ± 0.2 (%)	PD ≥ 4 mm (%) group 1 Max 12 m: 3 ± 0.1PD ≥ 4 mm (%) group 1 Mand 12 m: 3.6 ± 0.2PD ≥ 4 mm (%) Group 2 Max 12 m: 3.3 ± 0.1PD ≥ 4 mm (%) group 2 Mand 12 m: 4.1 ± 0.2PD ≥ 4 mm (%) group 1 Max 24 m: 2 ± 0.1PD ≥ 4 mm (%) group 1 Mand 24 m: 2.4 ± 0.2\PD ≥ 4 mm (%) group 2 Max 24 m: 1.8 ± 0.1PD ≥ 4 mm (%) group 2 Mand 24 m: 2.1 ± 0.2
6	Ibraheem et al. [20]	Egypt	*Bulletin of the National Research Centre*	Medical Excellence Centre, National Research Centre, Cairo	Randomized clinical study	6 and 12 months	Group 1: 10 patients ILGroup 2: 10 patients CL	20 male55–70	Glycated hemoglobin does not exceed 7.5%	Group 1: 20Group 2: 20	Zero	Overdenture (ball attachment)	100%		MBL group 1 M 6 m: 0.82 ± 0.11MBL group 1 D 6 m: 0.75 ± 0.09MBL group 1 M 12 m: 1.01 ± 0.11MBL group 1 D 12 m: 1.04 ± 0.04MBL group 2 M 6 m: 0.79 ± 0.11MBL group 2 D 6 m: 0.74 ± 0.08MBL group 2 M 12 m: 1.03 ± 0.12MBL group 2 D 12 m: 1.38 ± 0.23	N/A	
7	Juncar et al. [26]	Romania	*Journal of International Medical Research*	N/A	Prospective study	6 months	Group 1: 4 patients (moderate controlled DM2)	2 female2 male52–60	Group 1: HbA1c 7.05% (6.8–7.3%)	16	Zero	All-on-Four	100%		Healed site implants: 0.67 mm (0–1.5 mm)Postextraction alveoli or immediately adjacent: 1.36 mm (0–2.6 mm)	N/A	

DM1 = type 1 diabetes mellitus; DM2 = type 2 diabetes mellitus; HbA1c = glycated hemoglobin; IL = immediately loaded dental implants; CL = conventional loaded dental implants; MBL = marginal bone loss; CBL = crestal boneloss; BOP = bleeding on probe; PD = probing depth; Max = maxilla; Mand = mandible; m = months; M = mesial; D = distal; N/A = not available

**Table 2:** Technical information about the dental implant placement and loading

	Study	Technical information
1	Tawil et al. [24]	• Conventional implant treatment was done when bone volume was adequate; • Extraction was followed by immediate implant placement; • Loading was also applied when indicated • Periodontal therapy was applied, when indicated, before any implant treatment; • All implant surgeries and clinical measurements (BOP, PI, PD) were performed by the same calibrated operator (GT) • Two hundred fifty-five Brånemark implants (75 turned surface and 180 TiUnite; Nobel Biocare) were placed
2	Aguilar-Salvatierra et al. [16]	• All implants were placed level with the bone crest (Straumann Bone Level implants); • Length of 10–14 mm; • Diameter of 3.3–4.1 mm; • Implant stability was confirmed by resonance frequency analysis, measuring implant stability quotient (ISQ) (Osstell Mentor); • Insertion torque of over 35 N/cm and an ISQ value of at least 60 units; • All implants were placed in the upper maxillary in patients with a correct plaque control; • After surgery, patients were also asked to brush softly with chlorhexidine toothpaste
3	de Araújo et al. [27]	• Dental implants (NobelSpeedy, Brånemark System® MkII, MkIII, MkIV, Nobel Biocare AB); • Minimum insertion torque of 30 N/cm before the final seating of the implant; • For single teeth and fixed partial prostheses, the final abutments were inserted on the day of surgery; a provisional crown or prosthesis (screw-retained) was connected; • After 6 months, the patients received their definitive prosthetic reconstruction with full-ceramic crowns or fixed partial prostheses • For full-arch rehabilitations, provisional full-arch acrylic-resin prostheses with titanium cylinders were manufactured at the laboratory and delivered on the day of surgery • Definitive acrylic-resin prostheses were delivered, typically 6 months post-surgery
4	Al-Amri et al. [17]	• In all groups, patients received bone level Straumann Bone Level implants; • Lengths: 10–14 mm; • Diameters: 3.3–4.1 mm; • All implants were placed and immediately loaded at the level of crestal bone in the anterior maxilla; • Insertion torque of 35 N cm; • Oral hygiene instructions were given and the patients were advised to start rinsing with an essential-oil based mouthwash (Listerine Zero, Johnson & Johnson) twice daily for 2 weeks, after 24 h of surgery • Non-surgical periodontal therapy and oral hygiene instructions in each group, participants were enrolled in a 6 monthly periodontal/peri-implant maintenance program in which, full mouth scaling was performed around all natural teeth and implant surfaces using an ultrasonic scaler • Oral hygiene instructions regarding regular tooth brushing were given and patients were encouraged to floss the teeth and peri-implant surfaces daily
5	Al-Amri et al. [19]	• Straumann AG Bone-level platform-switched implants; • Diameters: 3.3–4.1 mm; • Lengths: 10–14 mm; • Groups 1 and 2: 30–35 N cm • Immediate load 2 days after implant placement; • Screw-retained provisional crowns, replaced with permanent crowns 6 weeks later; • Temporary abutments: RC Temporary Abutment, Straumann AG • Attached with a force of 25 N cm to hold the acrylic crowns • The prefabricated temporary crowns designed with narrow occlusal table were relined with a Bis-acrylic composite (Protemp II; 3 M ESPE); • The interproximal contacts were designed as broader contact areas to distribute the forces of mastication and provide support
6	Ibraheem et al. [20]	• Two dental implants were inserted in mandibular canine areas bilaterally using flapless technique (Nobel Speedy Groovy RP, Nobel Biocare); • Guided surgery with Software (Nobel Clinician, Nobel Biocare); • Anchor pins (Guided Anchor Pin w1.5 mm, Nobel Biocare); • Pressure-indicating silicone (Fit Checker, GC, Tokyo, Japan); • The instruments used were a drill (Guided Start Drill, Nobel Biocare), twist drills with diameters of 2.0, 2.8, 3.2, and 3.4 mm (Guided Twist Drill, Nobel Biocare), and a removable sleeve (Guided Drill Guide, Nobel Biocare) • Immediately after surgery and for 1 week, all patients were instructed to keep wearing their dentures 24 h/day except at bed time and time of denture cleaning • Concerning group I patients, within 48 h after surgery, the dental implants were immediately loaded using ball and socket attachments
7	Juncar et al. [26]	• Four implants were placed in the maxilla: two posterior implants (12 mm long and 3.5 or 4 mm in diameter) were placed at an angle of 35°; • Two anterior implants (10 mm long and 3.5 or 4 mm in diameter) were placed vertically; • The implants were placed bilaterally at the locations of maxillary second premolars and lateral incisors • For patients who required dental treatments, tooth extractions were performed and pathological periodontal tissue was curetted • Prosthetic stumps were applied bilaterally to the implants at angles of 35° posteriorly and 0° anteriorly • All dental implants were placed with a peak insertion torque of 50 N/m • Subsequently, the primary stability of each dental implant was analyzed by Resonance Frequency Analysis with the implant stability quotient (ISQ); • Implants with an ISQ ≥ 65 were considered satisfactory for immediate prosthesis placement; • For up to 24 h, healing caps were placed over the prosthetic stumps • Provisional rehabilitation with dental implant support was achieved by using provisional screw-retained acrylic restorations • Following insertion of the provisional prosthesis at 24 h after dental implant placement; • Postoperative monitoring patients were asked to return at 6 months after dental implant placement for the final prosthetic restoration

### Survival rate

The survival rate was reported or assumed based on the number of dental implants that did not fail or that were not removed during the follow-up. All seven included studies reported the survival rate [16, 17, 19, 20, 24, 26,27]. In five studies, no dental implants were lost during the follow-up, which means that the survival rate was 100% [6, 17, 19, 20, 24]. The other two studies had a survival rate percentage between 86.3 and 100%, and were the first percentage of an uncontrolled DM2 group [16].

### Success rate

There are different methods of evaluating the success rate of dental implants. Usually, the requirements regard peri-implant biologic measures such as MBL, BOP, PD, and others depending on the guideline. For this reason, the success rate was not assumed for any study. These data were collected if the author specifically reported it. Four studies reported the success rates, and all of them had a 100% rate [19,20, 24, 26].

### MBL, BOP, and PD

Marginal bone loss (MBL) was the most reported peri-implant data within the included studies. Only one study did not report it [24], and two studies reported it generically [26, 27]. Three studies showed data for bleeding on probing (BOP) and probing depth (PD) [16, 17, 19].

### Risk of bias

**Fig. 2:**
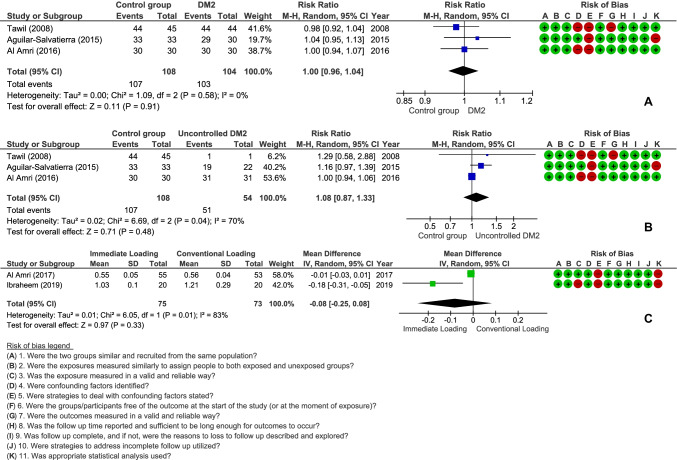
**a** Meta-analysis of survival of immediately loaded dental implants in individuals with controlled type 2 diabetes mellitus (DM2) in comparison with nondiabetic individuals. **b** Meta-analysis of survival of immediately loaded dental implants in individuals with uncontrolled type 2 diabetes mellitus (DM2) compared to nondiabetic individuals. **c** Meta-analysis of marginal bone loss in immediately loaded dental implants compared to conventional loading in type 2 diabetic patients

**Fig. 3:**
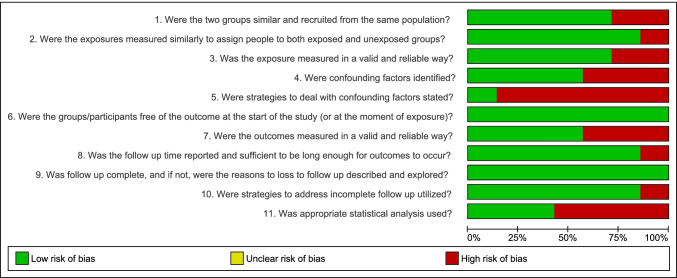
Risk of bias across included studies (Joanna Briggs Institute—Critical Appraisal Tool)

From the 5 studies included in the quantitative analysis [16, 17, 19, 20, 24], all of them were considered to a have low risk of bias, according to the JBI questionnaires (Fig. 2). The most problematic topics among the studies were the strategies to deal with confounding factors and the use of appropriate statistical analysis (Fig. 3).

### Quantitative analysis

All the studies included in the quantitative analysis had an IL DM2 and a control group, which consisted of nondiabetic patients or conventional loading of dental implants in DM2 (Table 1). The meta-analysis of IL in individuals with DM2 in comparison to nondiabetic individuals showed that there was no significant difference between the groups regarding the survival rate of dental implants (RR = 1.00, 95% CI 0.96–1.04; *p* = 0.91; *I*^2^ = 0%) (Fig. 2a), even if the patient had poor glycemic control (RR = 1.08, 95% CI 0.87–1.33; *p* = 0.48; *I*^2^ = 70%) (Fig. 2b). These first two meta-analyses included studies that followed the patients for at least 24 months. Meta-analysis of marginal bone loss in IL compared to conventional loading in DM2 patients after 12 months of follow-up also showed no significant difference between the groups (mean difference =  − 0.08, 95% CI − 0.25–0.08; *p* = 0.33; *I*^2^ = 83%) (Fig. 2c).

## Discussion

The definition of survival rate was consistent within all of the studies included in this systematic review, and it was elucidated as whether the dental implant was still in place at the time of the follow-up [16, 17, 19, 24, 25, 27, 28]. Controlled DM2 patients showed a remarkable survival rate of IL in all 7 studies, ranging from 90.5 to 100%. These qualitative results are supported by the meta-analysis, which reported no significant difference between IL in DM2 compared to nondiabetic patients. Since the 3 studies included in the meta-analysis followed the patients for at least 24 months, it is safe to make clinical decisions based on those results. IL presents a greater challenge to the immunologic system of DM2 patients, as it demands that the osseointegration process successfully occurs under surgical trauma, macro- and micromovements, and functional occlusion loading [16, 19, 29]. In DM2 patients, AGEs may permanently accumulate in the vessel walls, altering the phenotype of important cells such as macrophages, polymorphonuclear cells, fibroblasts, and endothelial cells [24]. Consequently, destructive inflammatory cytokines are produced, which leads to bone resorption around IL implants [17, 24]. The stiffness and modulus of elasticity of dental implants are much higher than those of the supporting bone; therefore, peri-implant vascularization is damaged due to chronic hyperglycemia. This biological process can collaborate negatively with the healing process, primarily at the coronal part of the bone in IL implants [20, 30].

Glycemic control was a critical and significant issue evaluated in all the included studies. The survival rate analysis regarding IL in uncontrolled DM2 compared to nondiabetic controls was also not statistically significant. The present results disagree with the majority of studies regarding the unsatisfactory outcomes of dental treatments in uncontrolled DM2 [31–33]. However, it is essential to consider that the 3 studies in the meta-analysis were heterogeneous in the methods, and this meta-analysis reported a high level of heterogeneity. Tawil et al. [24] included only one patient in the uncontrolled DM2 group, and this patient did not present implant failure. Aguilar-Salvatierra et al. [16] found a direct association between HbA1c and implant failures, considering that the uncontrolled group had a survival rate of 86.5% at the 24-month follow-up, compared to a 100% survival rate in the control group. In the third study included in the meta-analysis, the authors reported that the 31 uncontrolled DM2 patients enrolled in the study had a decrease in HbA1c levels during the 24-month follow-up. Satisfactory glycemic control in this study is directly associated with the high rate of implant survival [17]. Among other reasons, this association is probably correlated with the absence of bacteria and their products in systemic circulation [13, 17].

In the present systematic review, four studies reported a 100% success rate of IL in DM2 [19, 24–26]. The parameters used to assess the success rate were heterogeneous among the studies, which reveal the need for more evidence showing specific parameters for measuring the success rate of dental implants. Most of the studies classified the success rate based on the presence of peri-implant pathology, in which the diagnosis was based on MBL, BOP, and PD. Despite being composed of only 2 studies, the present meta-analysis comparing the MBL between IL and CL in individuals with DM2 after 12 months of follow-up showed no difference between the two techniques. These data contradict a meta-analysis of nondiabetic patients published in 2020 [34]. In their study, reduced crestal bone loss was found in conventional delayed loading of implants compared to immediately loaded implants.

When comparing peri-implant measures between individuals with DM2 and nondiabetic patients, the included studies had similar values for both groups. However, uncontrolled DM2 seems to present higher values of MBL, BOP, and PD [16, 17]. It is imperative to evaluate and ensure that the patient has satisfactory glycemic control, given that the HbA1c level is directly correlated with peri-implant pathology [21, 35]. The articles selected for inclusion in this systematic review agree upon the lack of differences in the success rate of implants with immediate loading in DM2 patients when compared to that of systemically healthy patients, even when the patient has poor glycemic control.

Hyperglycemia is a risk factor for advanced dental treatments that require a satisfactory inflammatory system [36–39]. The main oral issues associated with DM2 are the high incidence of carious lesions, higher prevalence of endodontic problems, and periodontal disease [7, 31, 40]. Thus, edentulism becomes one of the main consequences for these patients [6, 8]. Diabetic patients with uncontrolled glycemia have been shown to have worse results than those of controlled DM2 and nondiabetic patients, such as a higher incidence of implant failure [21, 32]. In addition to the fact that the disease is characterized by microvascular complications, tissue damage, and a higher risk of infection, these factors greatly influence dental treatment [5, 8, 41]. There are many published papers regarding the inverse association between poor glycemic control and dental treatment outcomes [42]. One of the major oral complications associated with success and survival rate in DM2 is the presence of vascular microangiopathy in the peri-implant tissues, such as alveolar bone and peri-implant mucosa [13, 21, 29, 43]. On the other hand, the influence of oral maintenance on HbA1c levels during the follow-up of dental implants in DM2 is a topic that is lacking information.

The most advanced techniques in dentistry are less studied in diabetic patients due to their potential risk of failure in these patients [24]. Many studies have proven that well-controlled diabetic patients with shorter disease onsets have preserved periodontal tissues and remarkably similar immunological responses to systemically healthy patients [44, 45]. Thus, it is safe to consider that techniques such as implants with immediate loading can be used safely in these patients [19]. There is a shortage in the literature regarding IL in DM2, especially concerning uncontrolled patients, since implant placement surgery is a relative or even absolute contraindication in these cases [46, 47].

Published studies thus far report divergent success rates in diabetic patients regarding conventional loading. A systematic review published in 2019 reports that DM2 individuals are more associated with a higher risk of peri-implant disease [32]. In contrast, another systematic review from 2016 proved that diabetic patients have similar success outcomes when compared to healthy patients [48]. However, the literature agrees that the major requirement to achieve success in implant placement surgery is satisfactory glycemic control pre- and postoperatively [32, 48, 49]. Multiple studies in the literature warn about the highest failure of dental implants in uncontrolled diabetic patients, regardless of the technique [32].

However, caution is necessary for any type of advanced technique in implantology in systemically compromised patients. The limitations of the present systematic review were primarily associated with the short number of studies published in the literature regarding the placement of immediately loaded implants in DM2. In addition, the heterogeneity of the methods also negatively influenced the analysis. The absence of important factors, such as the control group, glycemic control, survival rate, and success rate, of peri-implant analysis was also a problematic issue. The design of the included studies was different: longitudinal observational or clinical trials. Even though the starting point was the placement of an IL in DM2 and the outcome assessment was the same, the discrepancy in the design is a relevant limitation. The maximum number of studies included in the meta-analysis was three, which indicates the need for more studies to be published to increase the reliability of the results. Furthermore, with the exception of one study, the meta-analysis comprises only studies with a maximum follow-up of 24 months. This short period of time leads to uncertainty in the long-term results in the clinical practice of implantology. However, problems arising from an immediate loading protocol tend to occur in the first months during the osseointegration phase, so the two-year follow-up is adequate [50]. Nevertheless, to evaluate the survival rate, which can be influenced by peri-implant disease or crestal bone loss, these outcomes require long-term follow-up, and these results cannot be extrapolated in this study.

The results presented in this study are valid as long as all implant placement protocols for immediate loading are strictly followed. The articles also report the importance of oral hygiene habits and clinical prophylaxis periodically in these patients to assist in the maintenance of implants. The decision regarding MBL in IL should be carefully made, considering that the present meta-analysis is based on only 2 studies and that the previous systematic review published in 2020 was not able to conduct a meta-analysis. Thus, the survival of IL seems to be not related to type 2 diabetes mellitus. Higher peri-implant values or even the survival rate were associated with recurrent decompensated HbA1c levels, mostly in patients with poor oral hygiene or those who did not undergo peri-implant hygiene maintenance [16, 17, 28]. Peri-implant hygiene maintenance should be implemented in the general care of IL of DM2 patients, as it reduces hyperglycemia and promotes peri-implant health during the osseointegration period [16, 17, 19, 20, 28].

## Conclusion

The present study demonstrates that there is no difference in the survival of immediately loaded dental implants among nondiabetic individuals when compared to type 2 diabetic individuals, even when not controlled. When marginal bone loss in dental implants was compared between immediately loaded and conventional loading techniques in diabetic patients, there was also no significant difference. Thus, it is possible to affirm that if the clinician satisfactorily follows all surgical and prosthetic protocols, immediately loaded dental implants seem to be a safe treatment for individuals with type 2 diabetes, even for glycemic uncontrolled individuals. Oral hygiene is reported as an indispensable factor in the maintenance of these implants in diabetic patients. More original studies regarding the issue should be performed including more homogeneous studies so that it is possible to perform a systematic review.

## Supplementary Information

Below is the link to the electronic supplementary material.

Supplementary file1 (DOCX 18 KB)
