# Supplementary material for: Survival rate and peri-implant evaluation of immediately loaded dental implants in individuals with type 2 diabetes mellitus: a systematic review and meta-analysis
Source: Clin Oral Investig. 2021 Sep 29;26(2):1797–810. doi: 10.1007/s00784-021-04154-6 (PMC8479496; doi:10.1007/s00784-021-04154-6)
Supplement: Supplementary file 1 — Supplementary file1 (DOCX 18 KB) [file 784_2021_4154_MOESM1_ESM.docx]

| Database | Search (January 04^th^, 2021) | References (n) |
| --- | --- | --- |
| PubMed | ("diabetes mellitus"[All Fields] OR "diabetes mellitus type 2"[All Fields] OR ("hyperglycaemia"[All Fields] OR "hyperglycemia"[MeSH Terms] OR "hyperglycemia"[All Fields] OR "hyperglycaemias"[All Fields] OR "hyperglycemias"[All Fields] OR "hyperglycemia s"[All Fields]) OR "glucose intolerance"[All Fields] OR "noninsulin dependent diabetes mellitus"[All Fields] OR "systemic diseases"[All Fields] OR ("diabete"[All Fields] OR "diabetes mellitus"[MeSH Terms] OR ("diabetes"[All Fields] AND "mellitus"[All Fields]) OR "diabetes mellitus"[All Fields] OR "diabetes"[All Fields] OR "diabetes insipidus"[MeSH Terms] OR ("diabetes"[All Fields] AND "insipidus"[All Fields]) OR "diabetes insipidus"[All Fields] OR "diabetic"[All Fields] OR "diabetics"[All Fields] OR "diabets"[All Fields])) AND ("immediate dental implant loading"[All Fields] OR "dental implant"[All Fields] OR "dental implantation"[All Fields] OR "peri-implantitis"[All Fields] OR "immediately loaded implants"[All Fields] OR "peri-implant"[All Fields] OR "alveolar bone loss"[All Fields] OR "crestal bone loss"[All Fields] OR "peri-implant bone loss"[All Fields] OR "implant survival"[All Fields] OR "implant success"[All Fields] OR "implantology"[All Fields]) | 979 |
| Cochrane | ((“diabetes mellitus” OR “diabetes mellitus, type 2” OR hyperglycemia OR “glucose intolerance” OR “noninsulin dependent diabetes mellitus” OR “systemic diseases” OR diabetic)):ti,ab,kw AND ((“immediate dental implant loading” OR “dental implant” OR “dental implantation” OR “peri-implantitis” OR “immediately loaded implants” OR “peri-implant” OR “alveolar bone loss” OR “crestal bone loss” OR “peri-implant bone loss” OR “implant survival” OR “implant success” OR “implantology”)):ti,ab,kw | 105 |
| Scopus | ( TITLE-ABS-KEY ( ( "diabetes mellitus" OR "diabetes mellitus, type 2" OR hyperglycemia OR "glucose intolerance" OR "noninsulin dependent diabetes mellitus" OR "systemic diseases" OR diabetic ) ) AND TITLE-ABS-KEY ( ( "immediate dental implant loading" OR "dental implant" OR "dental implantation" OR "peri-implantitis" OR "immediately loaded implants" OR "alveolar bone loss" OR "crestal bone loss" OR "peri-implant bone loss" OR "implant survival" OR "implant success" ) ) ) | 1017 |
| Web of Science | TÓPICO: ((“diabetes mellitus” OR “diabetes mellitus, type 2” OR hyperglycemia OR “glucose intolerance” OR “noninsulin dependent diabetes mellitus” OR “systemic diseases” OR diabetic)) AND TÓPICO: ((“immediate dental implant loading” OR “dental implant” OR “dental implantation” OR “peri-implantitis” OR “immediately loaded implants” OR “peri-implant” OR “alveolar bone loss” OR “crestal bone loss” OR “peri-implant bone loss” OR “implant survival” OR “implant success” OR “implantology”)) | 557 |
| LIVIVO | KW=((("diabetes mellitus" OR "diabetes mellitus, type 2" OR hyperglycemia OR "glucose intolerance" OR "noninsulin dependent diabetes mellitus" OR "systemic diseases" OR diabetic) AND ("immediate dental implant loading" OR "dental implant" OR "dental implantation" OR "peri-implantitis" OR "immediately loaded implants" OR "peri-implant" OR "alveolar bone loss" OR "crestal bone loss" OR "peri-implant bone loss" OR "implant survival" OR "implant success" OR "implantology"))) | 727 |
| BVS (Portuguese and Spanish) | (("diabetes mellitus" OR diabetes OR hiperglicemia OR hiperglucemia OR "Intolerância à Glucose" OR "Intolerancia a la Glucosa") AND ("Carga Imediata em Implante Dentário" OR "Carga Inmediata del Implante Dental" OR "Implantes Dentários" OR "Implantes Dentales" OR "Perda do Osso Alveolar" OR "Pérdida de Hueso Alveolar" OR "Osseointegração" OR "Oseointegración")) | 55 |
| ProQuest | noft((("diabetes mellitus" OR "diabetes mellitus, type 2" OR hyperglycemia OR "glucose intolerance" OR "noninsulin dependent diabetes mellitus" OR "systemic diseases" OR diabetic) AND ("immediate dental implant loading" OR "dental implant" OR "dental implantation" OR "peri-implantitis" OR "immediately loaded implants" OR "peri-implant" OR "alveolar bone loss" OR "crestal bone loss" OR "peri-implant bone loss" OR "implant survival" OR "implant success" OR "implantology"))) | 22 |
| Open Grey | (("diabetes mellitus" OR "diabetes mellitus, type 2" OR hyperglycemia OR "glucose intolerance" OR "noninsulin dependent diabetes mellitus" OR "systemic diseases" OR diabetic) AND ("immediate dental implant loading" OR "dental implant" OR "dental implantation" OR "peri-implantitis" OR "immediately loaded implants" OR "peri-implant" OR "alveolar bone loss" OR "crestal bone loss" OR "peri-implant bone loss" OR "implant survival" OR "implant success" OR "implantology")) | 4 |
| Google Scholar | (("diabetes mellitus" OR hyperglycemia OR "noninsulin dependent diabetes mellitus" OR "systemic diseases") AND ("immediate dental implant loading" OR "dental implant" OR "immediately loaded implants" OR "bone loss" "implant survival" OR "implant success")) | 100 (3980) |
